# Non-Contrast-Enhanced Whole-Body Magnetic Resonance Imaging in the General Population: The Incidence of Abnormal Findings in Patients 50 Years Old and Younger Compared to Older Subjects

**DOI:** 10.1371/journal.pone.0107840

**Published:** 2014-09-26

**Authors:** Andrzej Cieszanowski, Edyta Maj, Piotr Kulisiewicz, Ireneusz P. Grudzinski, Karolina Jakoniuk-Glodala, Irena Chlipala-Nitek, Bartosz Kaczynski, Olgierd Rowinski

**Affiliations:** 1 2^nd^ Department of Clinical Radiology, Medical University of Warsaw, Warsaw, Poland; 2 Diagnostic Center, Medicover Hospital, Warsaw, Poland; 3 Department of Toxicology, Medical University of Warsaw, Faculty of Pharmacy, Warsaw, Poland; 4 Department of Medical Informatics and Telemedicine, Medical University of Warsaw, Warsaw, Poland; University of Modena & Reggio Emilia, Italy

## Abstract

**Purpose:**

To assess and compare the incidence of abnormal findings detected during non-contrast-enhanced whole-body magnetic resonance imaging (WB-MRI) in the general population in two age groups: (1) 50 years old and younger; and (2) over 50 years old.

**Materials and Methods:**

The analysis included 666 non-contrast-enhanced WB-MRIs performed on a 1.5-T scanner between December 2009 and June 2013 in a private hospital in 451 patients 50 years old and younger and 215 patients over 50 years old. The following images were obtained: T2-STIR (whole body-coronal plane), T2-STIR (whole spine-sagittal), T2-TSE with fat-saturation (neck and trunk-axial), T2-FLAIR (head-axial), 3D T1-GRE (thorax-coronal, axial), T2-TSE (abdomen-axial), chemical shift (abdomen-axial). Detected abnormalities were classified as: insignificant (type I), potentially significant, requiring medical attention (type II), significant, requiring treatment (type III).

**Results:**

There were 3375 incidental findings depicted in 659 (98.9%) subjects: 2997 type I lesions (88.8%), 363 type II lesions (10.8%) and 15 type III lesions (0.4%), including malignant or possibly malignant lesions in seven subjects. The most differences in the prevalence of abnormalities on WB-MRI between patients 50 years old and younger and over 50 years old concerned: brain infarction (22.2%, 45.0% respectively), thyroid cysts/nodules (8.7%, 18.8%), pulmonary nodules (5.0%, 16.2%), significant degenerative disease of the spine (23.3%, 44.5%), extra-spinal degenerative disease (22.4%, 61.1%), hepatic steatosis (15.8%, 24.9%), liver cysts/hemangiomas (24%, 34.5%), renal cysts (16.9%, 40.6%), prostate enlargement (5.1% of males, 34.2% of males), uterine fibroids (16.3% of females, 37.9% of females).

**Conclusions:**

Incidental findings were detected in almost all of the subjects. WB-MRI demonstrated that the prevalence of the vast majority of abnormalities increases with age.

## Introduction

Recent technological progress has enabled the implementation of whole-body magnetic resonance (WB-MR) screening in the general population. These technical developments include extended scanner table range, multiple input channels permitting parallel imaging, integrated high-resolution surface coils covering the whole body and high-quality sequences with short acquisition times [1]–[3]. Application of these hardware and software improvements facilitated a considerable reduction of the total imaging time without a significant decrease in image quality, which nowadays approaches the quality of dedicated examinations. Depending on the applied protocol, the imaging time of the whole body ranges from 20 to 90 minutes [1], [4]–[6]. Magnetic resonance imaging (MRI) not only has the high sensitivity required for the detection of abnormalities in different body organs including the head, spine, abdomen, pelvis and bones, but also offers the ability to characterize disease correctly in a significant number of cases, decreasing the number of false-positive diagnoses when compared to other currently used modalities, such as computed tomography (CT) or positron emission tomography (PET) [7], [8]. Due to its high sensitivity for the detection of tumor deposits, WB-MR imaging has been used effectively by many investigators in patients with different malignant diseases [8]–[14], however only a few centers have performed research on the utilization of this method for screening asymptomatic subjects [4]–[6], [ 15].

The main possible advantage of whole-body MRI in the general population is for the early detection of significant disease (e.g., malignant tumors, atherosclerosis) leading to rapid implementation of treatment and a likely improvement of the prognosis [2]. On the other hand, the shortcoming of such a screening is the depiction of a substantial number of incidental lesions, which cannot be accurately characterized based on WB-MR images [6], [5], [8], [15], [16]. These incidental findings often lead to the application of further investigation, not only generating additional costs, but also leading to severe psychological distress [17].

There is an additional and possibly beneficial aspect of whole-body imaging – MRI offers a unique opportunity to take an insight into the human body without considerable side effects. The majority of data regarding the prevalence of various abnormalities is based on anatomopathological postmortem studies. However in recent years, several papers, concerning incidental findings in patients undergoing different imaging examinations, including MRI of the brain and spine, have been published [18]–[24]. The current WB-MR techniques enable an estimation of the incidence of many abnormalities as well as – indirectly, by the analysis of their frequency in different age groups – an investigation of their natural history. Nevertheless, to date, no study analyzing the incidence of abnormalities found in WB-MR imaging in different age groups has been published.

Consequently, the aim of this study was twofold: firstly, to assess the incidence of significant abnormalities detected during non-contrast-enhanced WB-MR imaging; and secondly, to compare the incidence of abnormalities in two age groups: 50 years old and younger and over 50 years old.

## Materials and Methods

This study was approved by the academic bioethics committee. As the study was retrospective, a written consent was not obtained from the subjects included in this analysis. Patient records were anonymized and de-identified prior to analysis.

### Study population

The study group comprised 666 patients (465 male; 201 female) with a mean age of 46.4 years (age range 20–77 years), who underwent non-contrast-enhanced WB-MR screening at a private hospital between December 2009 and June 2013. The MRI examinations were contracted in accordance with special private health insurance, along with laboratory tests and other imaging studies, which were part of this screening program (chest X-ray, breast ultrasound or mammography, ultrasound of the abdomen and pelvis, cardiac CT for calcium score). All women underwent transvaginal ultrasound of the pelvis.

The inclusion criteria were as follows: age ≥18 years, willingness and ability to undergo MRI and participate in the screening. Exclusion criteria were contraindications to MRI (such as, peacemakers, metallic implants), severe claustrophobia, age <18 years, significant artifacts during MRI, precluding reliable assessment of obtained images. Since the aim of this study was to assess the incidence of abnormalities in general population (asymptomatic subjects), patients with significant clinical symptoms or with known, active disease at the time of MR imaging were also excluded from the analysis. Overall, 24 subjects were excluded from the analysis due to presence of clinical symptoms or known, active disease at the time of MR examination (n = 15), contraindications to MRI (n = 3), severe claustrophobia precluding MR scanning (n = 4), low quality of obtained MR images (n = 2).

There were 451 patients 50 years old and younger (the mean age 40.9 years) including 315 men and 135 women, and 215 patients over 50 years old (the mean age 57.9 years), among them 149 men and 66 women.

In 31 patients, the following surgical procedures were performed: emplacement of breast-implant devices (n = 9), cholecystectomy (n = 6), hysterectomy (n = 5), resection of the prostate (n = 3), reconstruction of the anterior cruciate ligament (n = 3), strumectomy (n = 2), mastectomy (n = 1), resection of the cerebellopontine angle tumor (n = 1), resection of liposarcoma of the lower extremity (n = 1). Malignant tumors were diagnosed in 9 patients prior to WB-MRI: uterine cancer (n = 2), cervical cancer (n = 1), ovarian cancer (n = 2), prostate cancer (n = 1), breast cancer (n = 1), liposarcoma (n = 1), thyroid cancer (n = 1). All of them underwent surgery and the interval between the operation and MR imaging in all cases exceeded 3 years. At the time of examination, none of these patients had clinical or laboratory evidence of the recurrence of neoplastic disease.

### MR imaging

Magnetic resonance imaging was performed on a 1.5-T system (Magnetom Avanto, Siemens Medical Solutions, Erlangen, Germany), using a phased-array multicoil system. The WB-MR imaging protocol consisted of the following sequences: T2-weighted short time inversion recovery (T2-STIR) of the whole body in the coronal plane, T2-STIR of the whole spine in the sagittal plane, T2-weighted turbo spin-echo (T2-TSE) sequence with fat-saturation of the neck and trunk in the axial plane, T2-weighted fluid attenuated inversion recovery (T2-FLAIR) of the head in the axial plane, 3D T1-weighted gradient recalled echo (3D T1-GRE) with fat saturation of the thorax in the coronal and axial planes, T2-TSE of the abdomen in the axial plane, 3D T1-GRE with fat saturation of the abdomen in the axial plane, chemical shift imaging of the abdomen in the axial plane. Selected parameters of the applied sequences are shown in table 1. The approximate examination time was 50 minutes.

**Table 1:** Selected parameters of applied MR sequences.

Parameter	T2 STIR	T2 STIR	T2 TSE fat-sat	FLAIR	3D T1 GRE	3D T1 GRE	T2 TSE	In-Out GRE
Imaged area	whole body	whole spine	neck-trunk	head	thorax	abdomen	abdomen	abdomen
Plane	coronal	saggital	axial	axial	axial, coronal	axial	axial	axial
TR/TE (ms)	6400/108	3000/50	2200/125	9000/92	3.24/1.24	4.89/2.23	2110/125	130/2.2/4.9
Flip angle (°)	150	150	150	150	10	10	150	70
Turbo factor	25	11	50	16	–	–	50	–
NSA	1	1	1	1	1	1	1	1
FOV (mm)	500×500	500×500	380×380	230×175	400×300	380×285	360×360	380×285
Matrix	384×346	320×320	256×256	256×192	288×156	288×147	256×256	256×173
Slice thickness (mm)	6	3	6	5	3.5	3	6	6
Number of sections	4	2	3	1	1 (in each plane)	1	1	1
Total acquisition time (all sections)	10 min	2 min 48 s	6 min 40 s	2 min 24 s	15 s (for each plane)	17 s	2 min 30 s	26 s

### Image analysis

The retrospective evaluation of all studies was performed by two radiologists with 15- and 10-years experience in interpreting MR images (and with 22 and 19 years of experience in general radiology, respectively), working at academic hospital. The assessment of WB-MR images was performed in MR reading room on a dedicated workstation. Readers were unaware of the patients’ clinical data. All abnormal findings were documented and classified according to the organ of origin and the significance. The criteria for lesion classification were determined before starting the assessment of MRI scans. Detected abnormalities were categorized as: of low significance, moderately or potentially significant, and significant. The abnormalities were classified as insignificant or of low significance (type I) if they did not require further evaluation or treatment (e.g., old brain infarcts, renal cysts, hepatic or splenic cysts or hemangiomas, disc herniations without compression of nervous structures, adrenal adenomas). The abnormalities were classified as moderately or potentially significant lesions (type II) if they required further medical evaluation, could cause clinical symptoms or required treatment (e.g., multiple sclerosis, gallstones, pulmonary nodules, spondylolisthesis ≥2°). A lesion was categorized as significant (type III) if it required immediate treatment or referral to verify its character.

In case of discrepancies between radiologists the final MRI diagnosis was based on consensus interpretation.

Then, a separate evaluation was performed in two different age groups: 50 years old and younger and over 50 years old to compare the prevalence of abnormalities in these subjects.

All WB-MR reports were delivered to referring primary care physicians, who decided upon further management.

### Follow-up and validation of significant imaging findings

The medical data of all subjects in whom significant abnormalities were demonstrated on WB-MR imaging was checked after, at least, a three-month interval following MRI. All information in terms of additional imaging, follow-up studies, implemented treatment and histopathological validation was recorded.

Overall, 695 of 3375 incidental findings (20.1%) reported by radiologists were confirmed, including 443 of 2997 type I lesions (14.8%), 237 of 364 type II lesions (65.1%) and all type III lesions (100%). In 5 patients with type III lesions, the reference standard for the diagnosis was histopathologic proof obtained intra-operatively. For 8 remaining type III lesions the confirmation was based on supplementary contrast-enhanced MRI, CT, ultrasound (abdominal and transvaginal) and follow-up studies.

Six hundred fifty-four of 680 confirmed type I and type II lesions were validated by other imaging studies, performed due to a private health insurance (chest X-ray, breast ultrasound or mammography, ultrasound of the abdomen and pelvis, cardiac CT for calcium score), whereas in 26 patients confirmation was based on the results of supplementary examinations (contrast-enhanced MRI, CT, ultrasound of the thyroid).

### Statistical analysis

The statistical analysis was performed using Statistica software (version 10.0).

A χ^2^ test was applied to compare the differences in the incidence of abnormalities (with the incidence of ≥1% in the studied population) between two groups: subjects 50 years old and younger and subjects over 50 years old. A *p*-value of <0.05 was considered significant.

## Results

The radiologists reported 124 different types of abnormalities. Overall, 3375 incidental findings were depicted in 659 (98.9%) of 666 subjects. Type I abnormalities (insignificant) constituted the highest number of incidental findings (n = 2997; 88.8%), followed by type II (moderately or potentially significant; n = 363; 10.8%) and type III lesions (significant; n = 15; 2.3%). Type I and type II lesions with an incidence of ≥1% in the screened population as well as their prevalence in the two different age groups (50 years old and younger and over 50 years old) are listed in tables 2 and 3.

**Table 2:** The incidence of the most frequent type I lesions in two age groups.

Region	Imaging finding	≤50 years: number (frequency)	>50 years: number (frequency)	Statistically significant difference*	All: number (frequency)
Brain	Lesions with brain infarct pattern	85 (18.8%)	84 (39.1%)	Yes (p<0.00001)	169 (25.4%)
	Lesions suggestive of leucoaraiosis	1(0.2%)	16 (7.4%)	Yes (p<0.00001)	17 (2.6%)
	Pineal gland cyst	10 (2.2%)	2 (0.9%)	No (p = 0.19)	12 (1.8%)
	Arachnoid cyst	4 (0.9%)	6 (2.8%)	No (p = 0.084)	10 (1.5%)
	Cerebral atrophy	0	8 (3.7%)	Yes (p = 0.001)	8 (1.2%)
	Choroid plexus cyst	5 (1.1%)	2 (0.9%)	No (p = 0.8)	7 (1.1%)
Head and neck	Sinus mucosal thickening	225 (49.9%)	103 (47.9%)	No (p = 0.11)	328 (49.2%)
	Thyroid nodules/cysts	38 (8.4%)	43 (20%)	Yes (p = 0.0002)	81 (12.1%)
	Nasal septum deviation	28 (6.2%)	11 (5.1%)	No (p = 0.4)	39 (5.9%)
	Fluid in mastoid process	18 (4%)	10 (4.7%)	No (p = 0.85)	28 (4.2%)
	Enlarged neck lymph nodes	16 (3.5%)	5 (2.3%)	No (p = 0.29)	21 (3.2%)
Spine	Degenerative spinal disease (all)	391 (86.7%)	211 (98.1%)	No (p = 0.27)	602 (90.4%)
	Degenerative spinal disease(small-moderate)	289 (64.1%)	111 (51.6%)	Yes (p<0.00001)	400 (60.1%)
	Degenerative spinal disease(significant)	102 (22.6%)	100 (46.5%)	Yes (p<0.00001)	202 (30.3%)
	Scoliosis	71 (15.7%)	46 (21.4%)	No (p = 0.2)	117 (17.6%)
	Schmorl’s nodes	65 (14.4%)	31 (14.5%)	No (p = 0.62)	96 (14.4%)
	Spondylolisthesis	36 (8%)	25 (11.6%)	No (p = 0.25)	61 (9.2%)
	Meningeal cysts	35 (7.8%)	25 (11.6%)	No (p = 0.073)	60 (9%)
	Lesion with vertebral hemangiomapattern	25 (5.5%)	13 (6%)	No (p = 1)	38 (5.7%)
	Lumbarization of S1	13 (2.9%)	16 (7.4%)	Yes (p = 0.0165)	29 (4.4%)
	End plate fracture	5 (1.1%)	12 (5.6%)	Yes (p = 0.0013)	17 (2.6%)
Thorax	Fibrosis/adhesions	17 (3.9%)	20 (8.7%)	Yes (p = 0.0102)	37 (5.6%)
	Enlarged thoracic lymph nodes	24 (5.5%)	8 (3.5%)	No (p = 0.25)	32 (4.8%)
	Breast cysts	16 (3.7%)	8 (3.5%)	No (p = 0.89)	24 (3.6%)
Abdomen/GI	Liver lesions suggestive of cysts/hemangiomas	105 (23.3%)	79 (36.7%)	Yes (p = 0.004)	184 (27.6%)
	Splenic lesions suggestive of cysts/hemangiomas	13 (2.9%)	6 (2.8%)	No (p = 0.76)	19 (2.8%)
	Cystic lesions of the pancreas	3 (0.7%)	9 (4.2%)	Yes (p<0.00001)	12 (1.8%)
	Enlarged spleen	9 (2%)	1 (0.5%)	No (p = 0.088)	10 (1.5%)
	Enlarged abdominal lymph nodes	4 (0.9%)	6 (2.8%)	No (p = 0.0849)	10 (1.5%)
Retroperitoneum	Renal cysts	74 (16.4%)	93 (43.3%)	Yes (p = 0.0032)	167 (25.1%)
	Perinephric inflammatory changes	3 (0.7%)	11 (5.1%)	Yes (p = 0.0005)	14 (2.1%)
	Adrenal lesion suggestive of adenoma	4 (0.9%)	5 (2.3%)	No (p = 0.16)	9 (1.4%)
Male pelvis	Enlarged prostate	16 (5.1%)	51 (34.2%)	Yes (p<0.00001)	67 (14.4%)
	Varicocele	19 (6%)	18 (12.1%)	No (p = 0.0537)	37 (8%)
	Epididymal cyst	20 (6.3%)	7 (4.7%)	No (p = 0.35)	27 (5.8%)
	Hydrocele testis	5 (1.6%)	3 (2%)	No (p = 0.82)	8 (1.7%)
Female pelvis	Nabothian cysts	45 (33.3%)	19 (28.8%)	No (p = 0.4)	64 (31.8%)
	Prominent pelvic veins	16 (11.9%)	4 (6.1%)	No (p = 0.15)	20 (10%)
Musculoskeletal	Extra-spinal degenerative disease	98 (21.7%)	140 (65.1%)	Yes (p<0.00001)	238 (35.7%)
	Joint effusion	54 (12%)	29 (13.5%)	No (p = 0.91)	83 (12.5%)
	Baker’s cysts	40 (8.9%)	27 (12.6%)	No (p = 0.29)	67 (10.1%)
	Non-cystic bone lesions (benign appearance)	32 (7.1%)	14 (6.5%)	No (p = 0.56)	46 (6.9%)
	Bone marrow edema	23 (5.1%)	9 (4.2%)	No (p = 0.42)	32 (4.8%)
	Solitary bone cyst	11 (2.4%)	3 (1.4%)	No (p = 0.3)	14 (2.1%)

* χ^2^ test was applied to compare the differences in the incidence of abnormalities.

**Table 3:** The incidence of the most frequent type II lesions in two age groups.

Region	Imaging finding	≤50 years: number (frequency)	>50 years: number (frequency)	Statistically significant difference*	All: number (frequency)
Thorax	Pulmonary nodule	22 (5%)	37 (16.2%)	Yes (p<0.00001)	59 (8.9%)
Abdomen	Hepatic steatosis	69 (15.8%)	57 (24.9%)	Yes (p = 0.0044)	126 (18.9%)
	Gallstones	14 (3.2%)	16 (7%)	Yes (p = 0.0247)	30 (4.5%)
Femele pelvis	Uterine fibroid	22 (16.3%)	25 (37.9%)	Yes (p = 0.0047)	47 (23.4%*)
	Ovarian cyst (>2 cm)	16 (11.9%)	4 (6.1%)	No (p = 0.15)	20 (10%*)
Vascular	Varicose veins	10 (2.3%)	7 (3.1%)	No (p = 0.53)	17 (2.5%)

* χ^2^ test was applied to compare the differences in the incidence of abnormalities.

**Figure 1:**
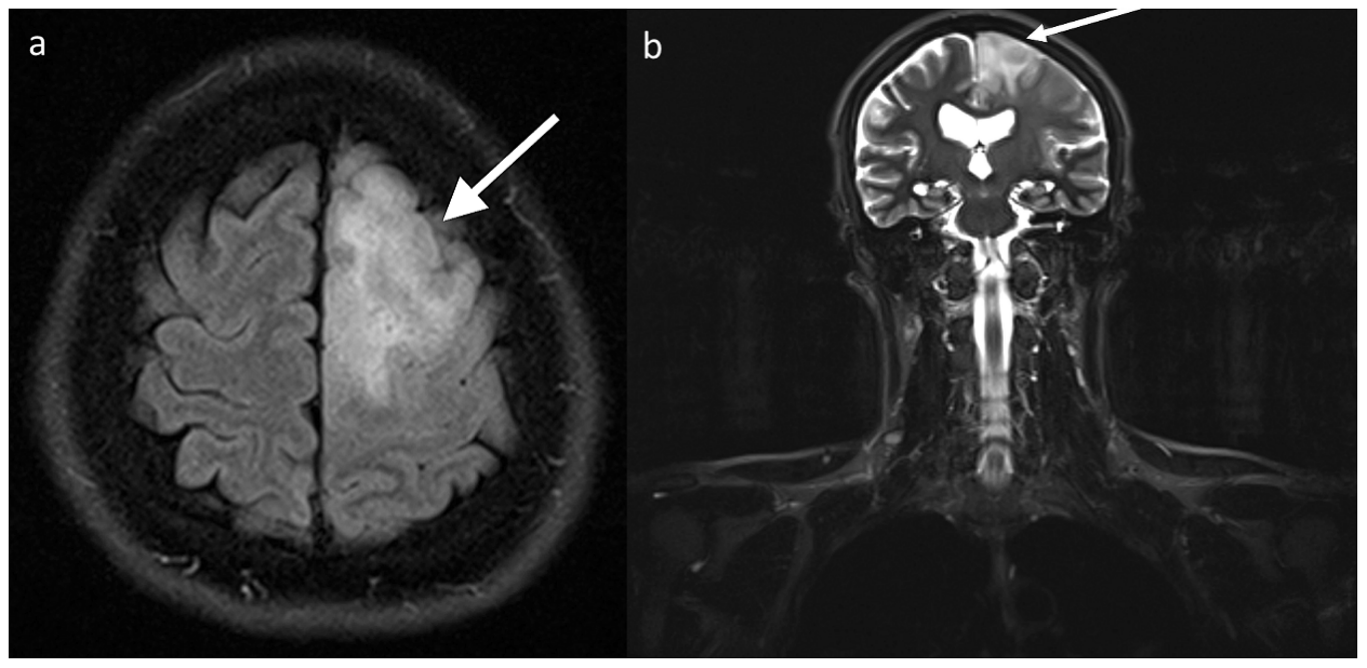
A 43-year-old male with cerebral glioma (arrows) confirmed histopathologically and excised. An axial T2-FLAIR image of the head shows a hyperintense area in the left frontal lobe (A). This lesion is also seen on a coronal whole-body T2-STIR image (B).

**Figure 2:**
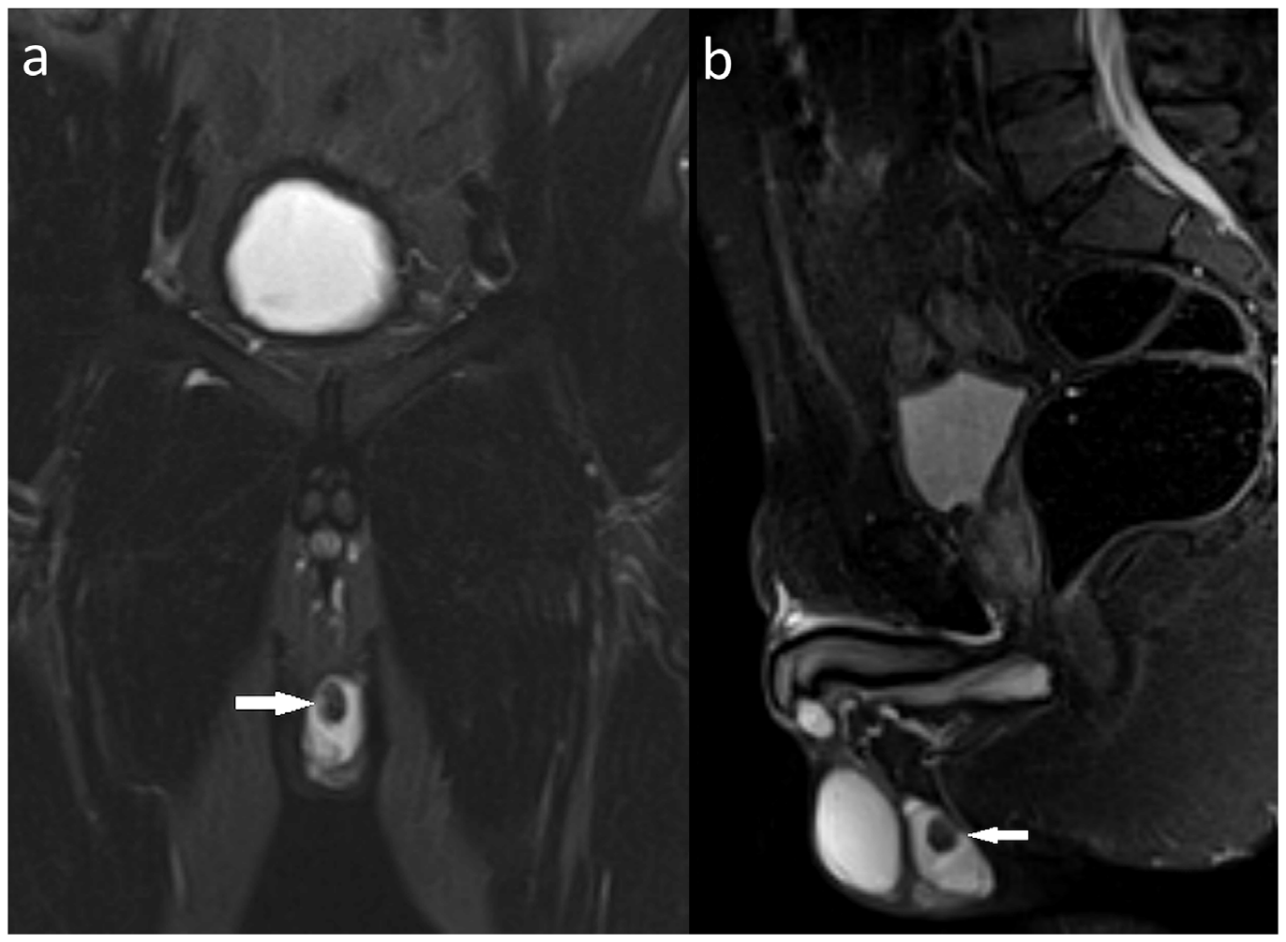
A 43-year-old male with confirmed Leydig cell tumor of the left testis seen as a hypointense lesion (arrows) on a coronal whole-body T2-STIR image (A) and a saggital whole-spine T2-STIR image (B).

**Figure 3:**
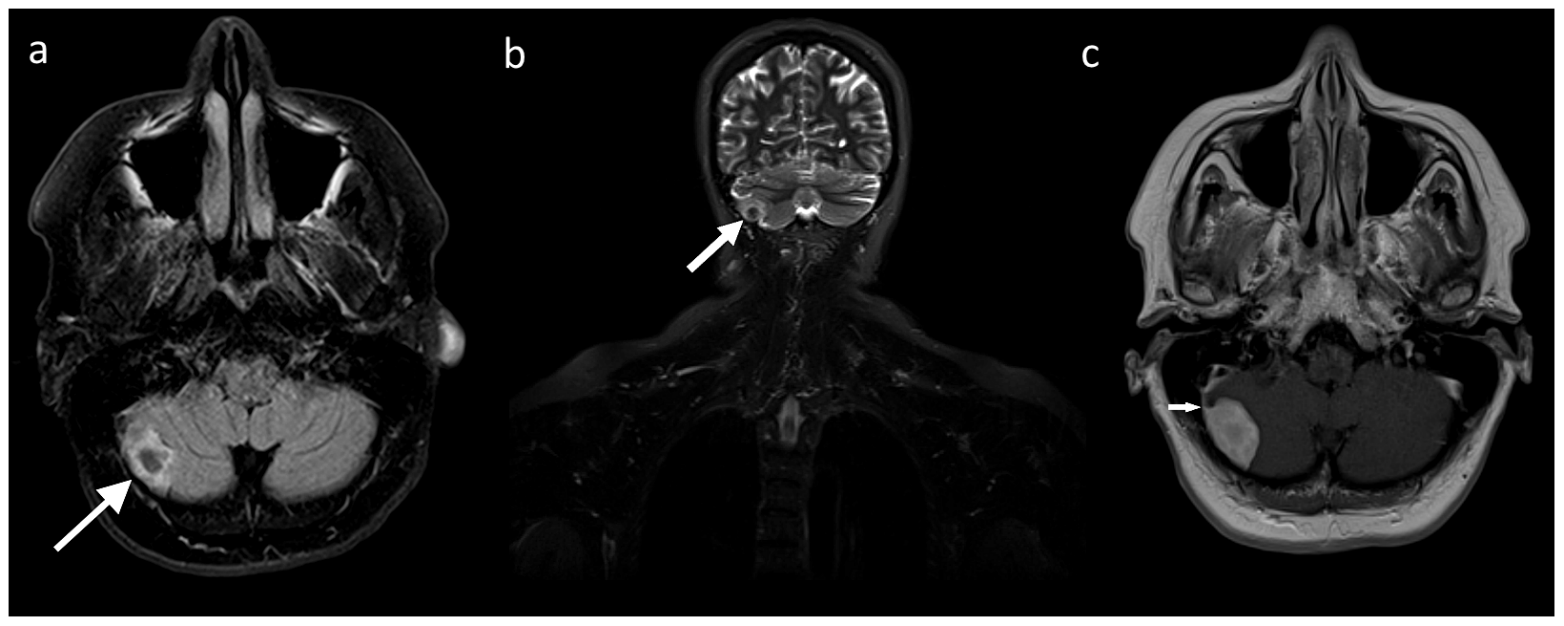
A 59-year-old female with surgically removed meningioma. A lesion of mixed signal intensity (arrows) is seen in the posterior fossa on a T2-FLAIR image of the head (A) and a coronal whole-body T2-STIR image (B). A supplementary, preoperative contrast-enhanced MR study (C) demonstrated enhancing lesion with a tail sign (arrow).

Fifteen lesions in 13 patients (2.0%) were categorized as significant (type III). Among them, there were nine types of malignant or possibly malignant lesions (in 7 patients) including 1 brain glioma (fig. 1), 1 bronchogenic carcinoma, 1 renal cell carcinoma, 1 complicated renal cysts (type 3 according to Bosniak classification), 1 ovarian tumor, 1 testicular Leydig cell tumor (fig. 2) and, in one patient, metastatic lesions in the lungs, liver and adrenal gland. Six benign lesions were also noted in this group: 3 meningiomas (fig. 3), 2 degenerative myelopathies and 1 lobar pneumonia. Five patients from this group were treated surgically (with glioma, bronchogenic carcinoma, renal cell carcinoma, Leydig cell tumor and meningioma), while in six of them (2 with meningiomas, 2 with degenerative myelopathy, 1 with metastatic disease and 1 with lobar pneumonia) conservative management was implemented. In the patient with metastatic lesions in lungs, liver and adrenal gland, no primary site of malignancy was identified and in another two patients (with ovarian tumor and complicated renal cysts) no medical data regarding management was available.

Analysis of the site of the depicted abnormalities revealed that 269 of them were located in the brain, 505 in the head and neck, 1072 in the spine, 102 in the lungs, mediastinum and breasts, 435 in the abdominal system and the gastrointestinal tract, 227 in the urinary tract, 91 in the male genital system, 157 in the female genital system, 494 in the musculoskeletal system and 23 in the cardiovascular system.

The most frequent (incidence >20%) were the following type I abnormalities: degenerative spinal disease (n = 602; 90.4%), sinus mucosal thickening (n = 328; 49.2%), hepatic cysts/hemengiomas (n = 184; 27.6%), brain infarcts (n = 169; 25.4%) and renal cysts (n = 167; 25.1%).

**Figure 4:**
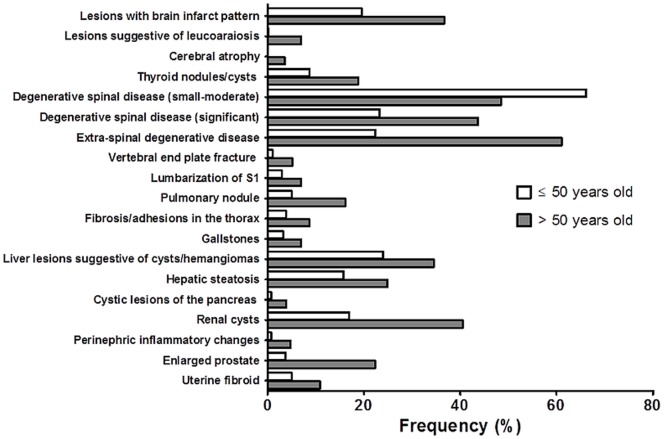
Graph illustrating the incidence of lesions, which reached statistically significant differences (p<0.05), in two different age groups.

A separate analysis performed in order to compare the prevalence of incidental findings in two different age groups revealed that the great majority of them was less frequent in the subjects 50 years old and younger as compared to subjects over 50 years old (tables 2 and 3) and the difference reached statistical significance for the following type I abnormalities: lesions with brain infarct pattern (18.8% vs. 39.1%, respectively), lesions suggestive of leukoaraiosis (0.2% vs. 7.4%), cerebral atrophy (0.0% vs. 3.7%), thyroid cysts/nodules (8.4% vs. 20%), small/moderate degenerative spinal disease (64.1% vs. 51.6%), significant degenerative spinal disease (22.6% vs. 46.5%), lumbalization of of S1 (2.9% vs. 7.4%), vertebral end plate fracture (1.1% vs. 5.6%), thoracic fibrosis/adhesions (3.9% vs. 8.7%), extra-spinal degenerative disease (21.7% vs. 65.1%), liver cysts/hemangiomas (23.3% vs. 36.7%), cystic lesions of the pancreas (0.7% vs. 4.2%), renal cysts (16.4% vs. 43.3%), perinephric inflammatory changes (0.7% vs. 5.1%), enlarged prostate (5.1% vs. 34.2% of males), as well as, for some type II lesions: pulmonary nodules (5.0% vs. 16.2%), hepatic steatosis (15.8% vs. 24.9%), gallstones (3.2% vs. 7.0%) and uterine fibroids (16.3% vs. 37.9% of females) (fig. 4).

Only some abnormalities, including pineal gland cysts, splenic enlargement and ovarian cysts, were more frequent in subjects 50 years old and younger than in the older population. However, the differences in the incidence of these lesions were not statistically significant.

## Discussion

The technical improvements in magnetic resonance imaging have enabled the application of this modality for the examination of the whole body. Obtaining both high contrast and high spatial resolution WB-MR images is now feasible within a reasonable acquisition time and the quality of the acquired images is approaching that of dedicated MR examinations [1], [5]. Moreover, there is no exposure to ionizing radiation, contrary to other imaging techniques which could be implemented for whole-body examination, such as computed tomography, positron emission tomography or scintigraphy.

Consequently, WB-MR imaging was used for the assessment of various neoplasms and, when compared to other whole-body imaging techniques, was advantageous with regard to the identification of bone marrow involvement, staging of tumors with low metabolic activity, detection of brain and hepatic metastases, lack of ionizing radiation and imaging of the pediatric population [8], [12], [25]. Other studies aimed at the assessment of preexisting non-neoplastic diseases, such as diabetes and arteriosclerotic vascular disease (ASVD), with whole-body contrast-enhanced magnetic resonance angiography (MRA) [26]–[28], musculoskeletal disorders, body fat distribution, or even postmortem findings [29]–[31].

Despite the various benefits of WB-MR imaging, to date only a few centers have carried out research on the implementation of this technique for screening asymptomatic subjects [4]–[6], [15]. Besides the economic aspects, one of the principal reasons of such caution is that whole-body screening of the general population with any modality still remains a controversial issue and the benefits of such imaging have never been proven [2], [17]. Thus, some authors advocate limiting the screening to certain groups, in which early detection of a disease or its complications may have potential clinical consequences and enable early and effective treatment (e.g., diabetes mellitus, advanced atherosclerosis, neoplasms) [2], [3], [26]–[28]. Regarding the two most lethal conditions affecting the general population (atherosclerosis, malignant tumors), the early detection of malignancy appears to be more important in terms of the implementation of urgent treatment and increased survival time (e.g., renal cell carcinoma, bronchial carcinoma) [2]. For that reason, our WB-MR imaging protocol focused more on tumor detection than on the identification of cardiovascular disease, however, the optimal method of whole-body screening is still a matter for discussion.

The cost of whole-body MR examinations is another crucial issue. For an extended, multi-organ study, approaching 60 minutes as in the case of our examinations, the cost is commonly related to the time of scanner occupation and is usually close to twice or three times the price of a standard MRI (e.g., head, spine). Moreover, the cost of the whole procedure should include the expense of additional tests, such as ultrasound, mammography, computed tomography, positron emission tomography, endoscopy, follow-up MRI, biopsies and laboratory tests, implemented to verify potentially relevant WB-MR imaging findings.

A further important consideration are the false-positive or undetermined MRI results. Despite the relatively high specificity of MRI for discrimination between benign and malignant lesions, there are a substantial number of cases, in which WB-MR imaging cannot confidently exclude malignancy. Incidental findings are a well-recognized problem in radiological screening programs [24], and the number of “incidentalomas” rises with an increase in the sensitivity of imaging modalities [22], [23], leading to substantial psychosocial distress [17]. Moreover, Schmidt et al. have found strong disagreement between patients’ and radiologists’ recognition of the severity of reported abnormalities [17]. Thus, even some insignificant findings (e.g., liver or splenic hemangiomas/cysts, renal cysts) may lead to severe mental distress for a patient.

Based on previous reports, as well as on our subjective impression after evaluating over 600 studies, we assume that the assessment of abnormalities by WB-MR imaging varies for different body regions and different pathologies [4]–[6], [15]. We presume that our scanning protocol of a non-contrast enhanced WB-MRI enabled the appropriate assessment of the majority of lesions in the brain, spine, paranasal sinuses, thyroid, lungs, abdominal parenchymal organs, adrenals, pelvis, testes and bones. Conversely, a detailed evaluation of the cardiovascular system, colon, joints, neck and prostate (except for its size and volume) could be compromised. Until recently, an important drawback of MRI was the compromised imaging of the lung. Currently, the improvement in MRI techniques, particularly the introduction of the 3D T1-GRE sequence, has helped to overcome this problem. According to published papers [32], [33], as well as to our own experience, this technique enables the detection of clinical relevant pulmonary nodules with high sensitivity.

To date, there are results available from four other groups imaging asymptomatic subjects with WB-MR. In three of these reports, the number of analyzed examinations was less than 300, thus our study group comprise the second largest number of subjects. Furthermore, we evaluated, for the first time, the prevalence of abnormalities in different age groups. The comparison of our results with the results of previous investigations is not straightforward, due to several factors including different MRI systems and protocols used for image acquisition, various methods of data analysis and a lack of standardization for the classifications of detected lesions [4]–[6], [15]. Despite quite similar criteria for determining the significance of depicted abnormalities (e.g., “relevant findings”, “might require further non-urgent evaluation or treatment”, “requires prompt medical follow-up”, “requires immediate referral”) the final categorization was to some extent subjective. Furthermore, two studies assessed the prevalence of cardiovascular disease, implementing, for that purpose, contrast-enhanced MRI [4], [6], whereas two other groups focused on detection of other abnormalities and used no contrast agent [5], [15].

The incidence of abnormalities detected in our group of subjects during WB-MR imaging (98.9%) was similar to that previously reported by Lo et al. and Hegenscheid et al. (>90%) [5], [6]. The much lower detection rate noted by Morin et al. (29.1%), was probably due to the use of only two sequences (T1-weighted SE sequence in the axial plane and T2-weighted GRE survey sequence), inclusion of a young cohort of subjects with a median age of 36 years and the lack of detailed imaging of the spine and brain [15]. We presume that the implementation in our imaging protocol of several devoted techniques enabled the acquisition of detailed images of the head, spine, lungs and abdomen, facilitating a significant improvement in the detection rate of some common abnormalities (e.g., cerebral infarcts, degenerative spinal disease, pulmonary nodules, liver cysts/hemangiomas, renal cysts, uterine fibroids) as compared to Morin et al.

A comparison of our findings with that of Goehde et al. is even more difficult as they focused on imaging of the brain, cardiovascular system and colon, using for this purpose contrast-enhanced WB-MR angiography, cine true-FISP technique and MR colonography [4]. Our scanning protocol did not comprise contrast-enhanced techniques, however, it included several sequences dedicated to spinal, thoracic and abdominal imaging. Consequently, the incidence of such abnormalities as degenerative spinal disease, pulmonary nodules (fig. 4), hepatic cysts/hemengiomas, splenic cysts/hemangiomas, was clearly higher among our group of subjects.

**Figure 5:**
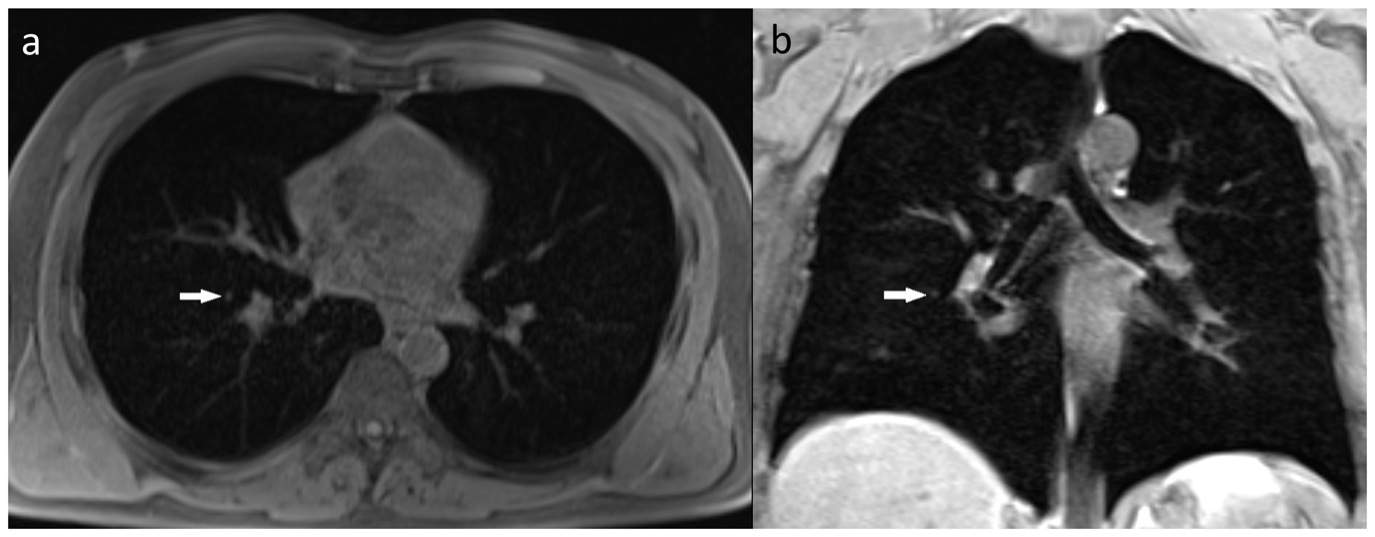
A 55-year-old female with a 5 mm nodule in the right lung (arrows). The lesion noted on both 3D T1-GRE axial (A) and coronal (B) images.

The WB-MR protocol implemented in the present study was in some aspects similar to that used by Lo et al. [5], in terms of the number and type of sequences. Therefore, the detection rates of the major abnormalities including T2 hyperintense lesions in the liver (24.0% vs. 34.5% in our study group) and renal cysts (18% vs. 25% in our study group) are comparable. However, we cannot exclude that the application of two T2-weighted abdominal sequences (T2-TSE, T2-TSE fat-sat in axial planes) may have resulted in slightly higher detection rates in our material. The higher difference noted in the detection rates of pulmonary nodules (8.9% in our group, compared to 3% in the group studied by Lo et al. [5]) was probably related to the implementation of 3D T1-GRE sequence in our imaging protocol (fig. 5). This sequence was effective in detecting even small pulmonary nodules and similar results have been obtained previously by researchers focusing on lung imaging [32], [33].

The protocol implemented in our study was shorter than that proposed by Hegenscheid et al. [6]. We omitted several techniques applied by Hegenscheid et al. for imaging of the brain (T2-TSE, T1-MPR, DWI, SWI, TOF angiography), thorax (T2-HASTE) and abdomen (3D MRCP, DWI). Thus, we could miss small aneurysms or other intracranial lesions, including small extra-axial meningiomas, as well as acute brain infarctions. On the other hand, we believe that the inclusion of the T2-FLAIR sequence, as in our study, facilitates detection of the majority of significant intra- and extra-axial lesions without the use of a contrast agent. As the incidence of intracranial aneurysms was only 0.2% on our WB-MR images, it is probable that due to the exclusion of the TOF MRA sequence from the protocol, we missed some of these lesions, as their reported incidence is 1.8–2.4% [6], [19]. Alternatively, omitting DWI could result in overlooking acute brain infarction, even though its incidence in the general population was very low (0.16%), according to Hegenscheid et al. [6]. Moreover, they are usually symptomatic.

**Figure 6:**
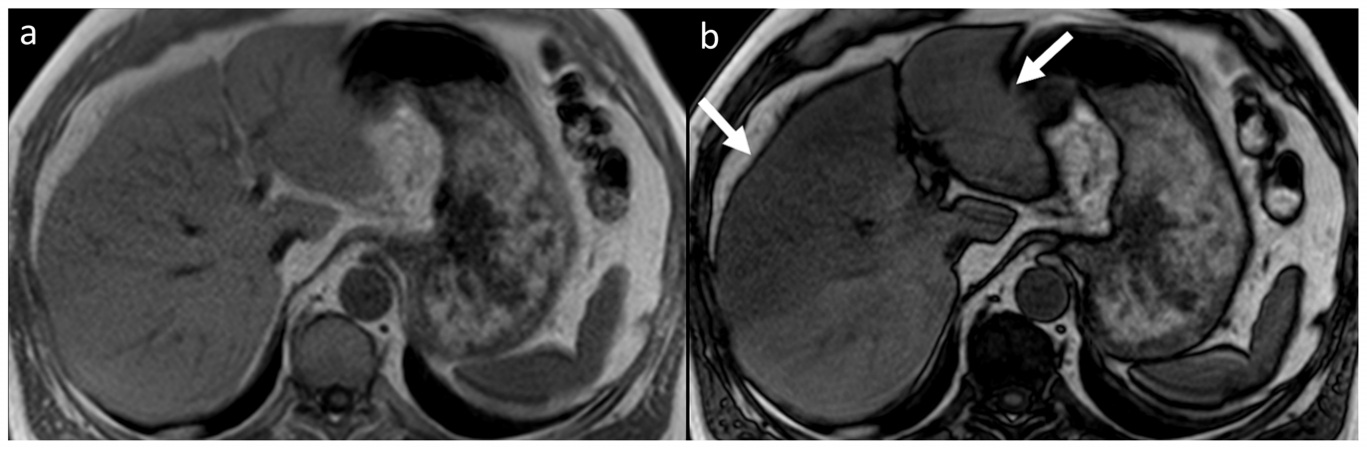
In-phase (A) and opposed phase (B) chemical shift MR images of a 51-year-old male demonstrate hepatic steatosis, consistent with areas of decreased signal intensity on an opposed-phase image. Steatosis is more pronounced it the left lobe and the anterior segments of the right liver lobe (arrows in B).

As compared to the abdominal protocol used by Hegenscheid et al., our assessment of the biliary tree could be compromised, however, the application of two axial T2-TSE sequences (without and with fat saturation) enabled the detection of dilated bile ducts and the visualization of gallstones in a relatively high number of subjects (n = 30, 4.5%). Implementation of the DWI sequence could probably slightly improve the detection rate of focal hepatic and extra-hepatic lesions, although at the expense of prolonged imaging time. Alternatively, we applied chemical shift imaging of the abdomen, allowing the diagnosis of hepatic steatosis (incidence of 18.9% in our study group) and leading to the recommendation of the implementation of a proper diet and a reduction in the patient’s cholesterol intake (fig. 6). This technique also facilitates the discrimination of adenomas (incidence of 1.4% in our study group) from other adrenal lesions and permits the detection of lipids in some hepatic tumors (hepatocellular carcinoma, hepatic adenoma, focal hepatic steatosis, hepatic angiomyolipoma, lipoma) as well as in extra-hepatic lesions (e.g., renal angiomyolipoma). In our material, it also assisted in confirming the diagnosis of benign pancreatic lipoma in two subjects.

We expect that the results of our study will add some valuable information towards the optimization of a WB-MR screening protocol. Such a protocol is always a compromise between the quality/number of images/sequences and the acquisition time. The results of our study support the inclusion of some dedicated MR techniques, such as T2-FLAIR (head), 3D T1-GRE (thorax) and chemical shift imaging (abdomen) into a WB-MR imaging protocol, along with whole-body and whole-spine sequences. We applied 2D FLAIR sequence with 5 mm slice thickness for imaging of the brain. The application of 3D FLAIR images would not only allow obtaining high-resolution images with isotropic voxel size of 1 mm, but also enable multi-planar imaging of the head devoid of cerebro-spinal fluid flow artifacts often visible on 2D images [34]. This would facilitate detection of higher number of lesions in cerebral hemispheric white matter and brainstem, however on the expense of prolonged both acquisition time and time needed for the evaluation of images.

Additional factors which may influence the protocol of WB-MR screening are the aims of the investigation (cardiovascular screening, tumor detection or both), the general type of the examined population, and the individual characteristics of the participants. Screening of diabetic patients or subjects with increased risk of ASVD may lead to the application of whole-body MR angiography. Taking into consideration individual factors, such as body mass index (BMI) and family history of neoplastic disease, may lead to further personalization of the scanning protocol. In obese subjects application of Dixon technique facilitates segmentation of adipose tissue enabling its quantification and assessment of distribution [35]. Patients with known or suspected neoplasms as well as individuals with the family history of certain neoplasm require application of MR techniques sensitive for tumor detection, such as diffusion weighted imaging (DWI) [13], [36]. In addition, the use of short-tau inversion recovery (STIR) sequence in patients with multiple myeloma or bone metastases enables identification of bone marrow involvement and increases the sensitivity for the detection of bone lesion [37], [38].

The results of our study showed that non-contrast-enhanced WB-MR imaging facilitated the assessment of the incidence of numerous abnormalities in the general population and enabled the detection of significant findings in 2% of the patients, including 7 patients with malignant lesions (4 of whom were treated surgically). Even though the implemented imaging was beneficial for several subjects, the overall cost of WB-MR screening is high and might not be justified by the early detection of malignancy in a relatively small percentage of the scanned population.

The role of imaging modalities in the estimation of prevalence of various abnormalities considerably increased in recent years, mostly due to a significant increase in spatial resolution of CT and MRI. Based on imaging findings, the prevalence of a wide range of incidental findings such as silent brain infarcts, intervertebral disc degeneration, lung nodules, liver hemangiomas, adrenal adenomas, renal cell carcinomas and uterine fibroids, appeared to be more frequent than previously thought [18]–[24], [39]–[41].

A good example is renal cell carcinoma (RCC), which accounts for 90% of primary renal neoplasms and represents 3% of all adult malignancies [39], [40]. The incidence of this neoplasm has gradually risen during recent decades, mostly due to advances in imaging. Nowadays a classic clinical presentation triad of RCC (hematuria, flank pain, palpable abdominal mass) is seen in only 5–10% of cases. More than 60% of RCCs are now diagnosed incidentally by CT, MRI or ultrasound [40]. Since the majority of detected RCCs are currently smaller and of lower stage the number of partial or “nephron-sparing” nephrectomies has increased as compared to total nephrectomies. In addition, survival has improved, with an overall 5-year survival rate of 62%. The 5-year survival in higher for incidentally detected tumors (85%) than for symptomatic cases (53%) [40].

Solitary pulmonary nodule (SPN) is another abnormality, which occurs more frequently than previously thought. While it is found incidentally in only 0.09–0.2% of all chest radiographs, the reported incidence of SPN on CT scans is much higher, ranging from 8% to 51% [41]–[43]. The radiologic features of SPN, such as calcification or fat within a nodule, its size, margins, location and rate of growth help to determine the likelihood of malignancy in a majority of cases [41], [44]. Nevertheless, the American College of Physicians recommended CT follow-up at 3, 6, 12, and 24 months for indeterminate SPN [44].

Monitoring SPNs and other suspicious lesions detected incidentally on imaging studies might lead to better outcomes by detecting early cancers, but on the other hand diagnosis of indeterminate mass often results in severe distress for a patient and additional imaging and follow-up.

A novel aspect of our study was the use of magnetic resonance imaging for the analysis of the occurrence of abnormalities in different age groups, which could assist in a better understanding of their natural history. We noted a higher incidence of the great majority of abnormal lesions, such as, vascular infarcts, significant degenerative spinal and extra-spinal disease, pulmonary nodules, liver cyst/hemangiomas, gallstones, renal cysts and uterine fibroids, in subjects over 50 years old. However, we observed several interesting exceptions, including pineal gland cysts, splenic enlargement and ovarian cysts (>2 cm), which were more frequent in subjects 50 years old and younger, although, in both cases, the differences were not statistically significant. Moreover, in this population, we noted a significant incidence (>10%) of some abnormalities presumed to be typical for older subjects (e.g., vascular infarcts, substantial degenerative spinal disease, renal cysts). As the natural history of many lesions is not fully known, the detailed statistical analysis of WB-MR data of large cohorts of asymptomatic subjects in narrow age groups, concerning not only the prevalence of different abnormalities, but also their number and size, may provide some valuable information without the implementation of invasive techniques or postmortem studies.

This study has certain limitations. Firstly, the subjects were not selected randomly, but had WB-MR examinations due to private health insurance, which, in general, reflected their better material status as compared to the average population. Therefore, we cannot exclude that their average health status may somewhat differ from that of randomly selected subjects. In addition, there is an important ethical consideration – if WB-MR screening proves to be feasible then it cannot be restricted to a group of individuals but it has to be accessible to all people independent of their socio-economic status [2].

Secondly, the presence and the character of the majority of incidental findings noted during WB-MR imaging (79.9%) was not confirmed by other tests and the final conclusion was based on the subjective opinion of the radiologist evaluating the study. For this reason, we could not assess the number of false-negative cases and the true sensitivity of the implemented MR technique for detecting abnormalities in various organs, nor determine its specificity for discrimination between different lesions. All abnormalities classified as significant (type III) underwent further comprehensive assessment, while the whole studied population underwent supplementary imaging being a part of their private health insurance (chest X-ray, breast ultrasound or mammography, ultrasound of the abdomen and pelvis, cardiac CT for calcium score), which supported the verification of a number of diagnoses (e.g., hepatic steatosis, gallstones, urinary tract calculi, uterine fibroid, prostatic enlargement). Even though, the majority of incidental findings was not validated. As this is a natural limitation of similar studies, investigators undertaking analogous research experienced the same problem [5], [6], [15].

Thirdly, the comparison of the prevalence of abnormalities in two different age groups should be regarded as a preliminary. For a more comprehensive evaluation, further studies are needed based on even larger number of subjects.

In a current study the use of relatively new technical applications such as extended scanner table range, parallel imaging combined with multiple input channel, high-resolution surface coils covering the whole body (total imaging matrix, TIM) and sequenced with short acquisition time enabled us to obtain high quality images of the whole body within acceptable time. We presume that in the future the role of high field systems (≥3T) for whole-body MR imaging may increase since they offer substantially higher signal-to-noise ratio (SNR) and contrast-to-noise ratio (CNR) than lower field systems. Increases in SNR and CNR can result in improving image resolution and shortening time of imaging [45]. However there are several potential drawbacks of imaging at high-field scanners, including limitation of energy deposition, increased magnetic susceptibility and chemical shift artifacts, marked increase in specific absorption rate (SAR) and problem with radio frequency (RF) and field inhomogeneity [45]. These technical limitations especially apply to imaging larger fields of view and for thoracic and abdominal scans may result in significant image distortion and signal loss related to soft-tissue and gas interfaces (e.g. bowel gas in the abdomen and pelvis, lung bases in the upper abdomen). Moreover, the cost of installation and unkeep of high-field scanners is substantially higher compared with 1.5T units [45].

There are other relatively new MRI techniques which could be used for a variety of MR imaging, such as functional magnetic resonance imaging (fMRI), diffusion tensor imaging (DTI), voxel based morphometry (VMB), positron emission tomography combined with magnetic resonance imaging (PET-MRI) or traveling wave MRI [46], [47]. Functional MRI enables the indirect (based on hemodynamics) measurement of brain activation, DTI allows identification of white matter tracks (fiber tracking) in the brain and spine, which can be displayed as DTI maps, VBM facilitates monitoring changes in gray matter (related to aging, drug abuse, psychiatric disorders or other environmental and health factors) and PET-MRI, which yields information about biochemical processes, permits detection and staging of neoplastic diseases [46]. However, the above techniques are more suited for dedicated MR examinations and its role for screening in asymptomatic subjects currently appears to be limited. Traveling wave MRI is an interesting new technique, which relies on traveling radio-frequency waves sent and received by an antenna, allows more uniform coverage of samples that are larger than the wavelength of the MR signal [47]. Despite uniform coverage of larger volumes this technique requires very high magnetic fields and at this time cannot be implemented for whole-body screening [47].

In conclusion, the results of our study confirmed that non-contrast-enhanced WB-MR imaging is a reliable, safe and accurate method enabling the detection of disease throughout the entire body, including malignant tumors. They also support the incorporation of some dedicated MR techniques, in particular T2-FLAIR, 3D T1-GRE and chemical shift imaging into the screening protocol. The evaluation of the prevalence of abnormalities in different age groups showed that most of them increase with age. We believe that in the future such analyses may have an important cognitive aspect and facilitate the understanding of the natural history of different pathologies, especially their incidence and growth rates.
